# Smoking in Asthma Is Associated with Elevated Levels of Corticosteroid Resistant Sputum Cytokines—An Exploratory Study

**DOI:** 10.1371/journal.pone.0071460

**Published:** 2013-08-09

**Authors:** Mark Spears, Charles McSharry, Rekha Chaudhuri, Christopher J. Weir, Carl de Wet, Neil C. Thomson

**Affiliations:** 1 Respiratory Medicine, Institute of Infection, Immunity and Inflammation, University of Glasgow, Glasgow, United Kingdom; 2 Immunology, Institute of Infection, Immunity and Inflammation, University of Glasgow, Glasgow, United Kingdom; 3 Robertson Centre for Biostatistics, University of Glasgow, Glasgow, United Kingdom; 4 Edinburgh Medical Research Council Clinical Trials Methodology Hub, Public Health Sciences, University of Edinburgh, Edinburgh, United Kingdom; 5 NHS Education for Scotland, Glasgow, United Kingdom; University Hospital Freiburg, Germany

## Abstract

**Background:**

Current cigarette smoking is associated with reduced acute responses to corticosteroids and worse clinical outcomes in stable chronic asthma. The mechanism by which current smoking promotes this altered behavior is currently unclear. Whilst cytokines can induce corticosteroid insensitivity *in-vitro,* how current and former smoking affects airway cytokine concentrations and their responses to oral corticosteroids in stable chronic asthma is unclear.

**Objectives:**

To examine blood and sputum cytokine concentrations in never, ex and current smokers with asthma before and after oral corticosteroids.

**Methods:**

Exploratory study utilizing two weeks of oral dexamethasone (equivalent to 40 mg/day prednisolone) in 22 current, 21 never and 10 ex-smokers with asthma. Induced sputum supernatant and plasma was obtained before and after oral dexamethasone. 25 cytokines were measured by multiplex microbead system (Invitrogen, UK) on a Luminex platform.

**Results:**

Smokers with asthma had elevated sputum cytokine interleukin (IL) -6, -7, and -12 concentrations compared to never smokers with asthma. Few sputum cytokine concentrations changed in response to dexamethasone IL-17 and IFNα increased in smokers, CCL4 increased in never smokers and CCL5 and CXCL10 reduced in ex-smokers with asthma. Ex-smokers with asthma appeared to have evidence of an ongoing corticosteroid resistant elevation of cytokines despite smoking cessation. Several plasma cytokines were lower in smokers with asthma compared to never smokers with asthma.

**Conclusion:**

Cigarette smoking in asthma is associated with a corticosteroid insensitive increase in multiple airway cytokines. Distinct airway cytokine profiles are present in current smokers and never smokers with asthma and could provide an explanatory mechanism for the altered clinical behavior observed in smokers with asthma.

## Introduction

Cigarette smoking reduces acute treatment responses to corticosteroids [1], [2], [3], [4], [5], [6], [7], [8], [9], and is associated with increased symptoms [10], [11] and unscheduled emergency department visits in patients with asthma [12]. A long lasting effect of former smoking on the therapeutic effects of corticosteroids in asthma is also suggested by the attenuated responses found in ex-smokers with asthma with significant smoking histories [8], [9]. As a history of current or past smoking is present in approximately half of subjects with asthma, cigarette smoking could be viewed as one of the most important environmental factors influencing this condition.

Whilst the impact of smoking on airway inflammation in asthma has become clearer over recent years, the mechanism/s by which cigarette smoking reduces corticosteroid responses in asthma is still unknown. Alteration of airway cytokine profiles in response to current and former cigarette smoking is a plausible candidate mechanism as previous research has demonstrated that combinations of cytokines can alter corticosteroid responsiveness *in-vitro* [13], [14], [15], [16]. Increased concentrations of sputum supernatant interleukin (IL)-8 (CXCL8) [17], endobronchial interferon (IFN)-γ [18] and reduced sputum supernatant IL-18 [19] have been observed in smokers with asthma, leading us to hypothesize that cigarette smoking induced alterations in the airway cytokine environment in asthma could be contributing to the reduced acute airway responses to corticosteroids observed in this group.

Therefore we measured a broad selection of cytokines in sputum supernatant and plasma in a group of subjects with asthma with differing smoking histories, both before and after an oral corticosteroid trial to gain insights into the effect of current and former smoking on cytokine profiles and their responses to corticosteroids in asthma.

Our results suggest that cigarette smoking promotes a corticosteroid insensitive alteration in airway inflammation in asthma which may be responsible for the altered clinical phenotype in current smokers with asthma and that long term cessation of smoking does not restore airway cytokine expression to the pattern observed in never smokers with asthma. Given the frequency of current and former cigarette smoking in asthma our findings potentially have implications for both current approaches to treatment and the development and licensing of new treatments for adults with stable asthma. Further research in this area is required to examine and extend the findings presented herein.

## Methods

### Subjects and Study Design

Three groups of patients, (i) current smokers, (ii) ex-smokers and (iii) never smokers with stable asthma were recruited from hospital out-patient clinics, general practices and previous studies to an exploratory study with open label, unblinded use of oral dexamethasone. Our exclusion criteria were: requirement for treatment with or the presence of conditions likely to be exacerbated by oral corticosteroids, intention to stop smoking or become pregnant, current pregnancy or any additional major chronic illness. Treatment with up to a maximum of 2000 µg beclometasone (or equivalent) per day, long acting beta_2_ agonists and leukotriene receptor antagonists was allowed. In an attempt to guide study design we performed a power calculation based on previously published results [8]. Using this information we estimated that if twenty subjects were recruited to both the smokers and never smokers with asthma groups would could expect to have 80% power to detect a between group difference of at least 336.4 ml in pre-bronchodilator FEV_1_ in response to the oral dexamethasone trial.

### Ethics Statement

All subjects provided written informed consent prior to participation and the study was reviewed and approved by the West of Scotland ethics committee.

We defined stable asthma as no emergency clinic or hospital visit, oral corticosteroid prescription or change in asthma treatment in the preceding month. Current smoking was defined as ≥5 cigarettes/day and ≥5 pack year history. Ex-smokers with asthma were eligible if they had ceased smoking two or more years from the date of recruitment, were former daily smokers of ≥5 cigarettes and had a ≥5 pack year history. ‘Never smoking’ subjects were required to have no history of regular daily smoking and to be current non-smokers. All subjects performed urine cotinine (SmokeScreen™ sampling system, GFC Diagnostics, UK) and exhaled carbon monoxide testing (Pico Smokerlyser®, Bedfont Scientific Ltd, UK) at each visit to confirm smoking status. Smokers were required to abstain from smoking for at least three hours prior to tests whilst inhaled bronchodilator was withheld as per international guidelines for testing. Inhaled corticosteroids were withheld for twenty four hours prior to testing.

Eligibility for the study required demonstration of reversible airflow obstruction [FEV_1_ bronchodilator response to β_2_ agonist of ≥12% and >200 ml], peak expiratory flow (PEF) lability or a positive methacholine challenge test. All lung function assessments met international consensus guidelines [20], [21], [22]. Current and ex-smokers with asthma were required to have an FEV_1_>80% predicted before entry by methacholine challenge testing (to reduce confounding with chronic obstructive pulmonary disease). Airway corticosteroid sensitivity was assessed by change in pre-bronchodilator FEV_1_ to a two week trial of oral dexamethasone (6 mg/1.74 m^2^; roughly equivalent to 40 mg prednisolone/day). On the day prior to and on completion of the dexamethasone course all subjects performed spirometry, exhaled nitric oxide measurement at 50 ml/sec (Niox Flex, Aerocrine AB, Sweden), induced sputum and completed asthma control questionnaires (ACQ) [23]. Plasma was obtained by venepuncture to allow measurement of cytokines before and after the corticosteroid trial. Serum cortisol was assessed on completion of the dexamethasone course, subjects were deemed to have been compliant if their blood cortisol level was below 50 nmol/l.

### Sputum and Plasma Cytokine Measurement

Sputum samples were processed using the whole sputum sample method [24]. Briefly, homogenization was achieved using gentle mechanical disruption in low concentrations of dithiothreitol (0.05%). Samples with oral epithelial counts >80% were discarded. Sputum supernatants were collected and stored in aliquots at -80°C until processing. Plasma was collected from heparinised blood samples. Sputum supernatant and plasma was examined for mediators informative of pro and anti-inflammatory functions and Th1, Th2 and Th17 responses relevant to asthma using a multiplex immuno-detection system (25-plex cytokine assay, Invitrogen Ltd, UK) and a Luminex 100™ analyzer (Luminex Corporation, USA). The cytokines detected [assay kit detection limit in pg/ml] were interleukin (IL)-1 receptor antagonist (IL1RA) [10], IL-1β [18], IL-2 [6], IL-2 receptor (IL2R) [24], IL-4 [5], IL-5 [3], IL-6 [3], IL-7 [10], IL-10 [5], IL-12 (p40/p70) [5], IL-13 [10], IL-15 [10], IL-17 [16], interferon (IFN)α [10], IFNγ [5], granulocyte macrophage colony-stimulating factor (GM-CSF) [5], tumor necrosis factor (TNF)-α [5], C-X-C motif chemokine ligand (CXCL)8 [3], CXCL9 [6], CXCL10 [5], CC motif chemokine ligands (CCL)2 [10], CCL3 [10], CCL4 [10], CCL5 [15] and CCL11 [5]. Values that could not be confidently calculated were assigned a value half-way between the manufacturers lowest recommended detection level and zero to allow for statistical examination via logarithmic transformation. Plasma cytokines were not assessed in ex-smokers with asthma.

### Statistical Analysis

Between group comparisons were performed for smokers and never smokers with asthma only. Normal and non-normal distributed variables were analyzed using t-tests and Mann-Whitney U test respectively. For within group before/after dexamethasone treatment comparisons, paired t-test and Wilcoxon signed rank test were applied. Subjects’ post-corticosteroid data were utilized if there was evidence of compliance (as assessed by serum cortisol levels). Correlations were performed using Pearson’s correlation. Adjustment for multiple comparisons was not performed as all analyses were treated as exploratory.

**Figure 1:**
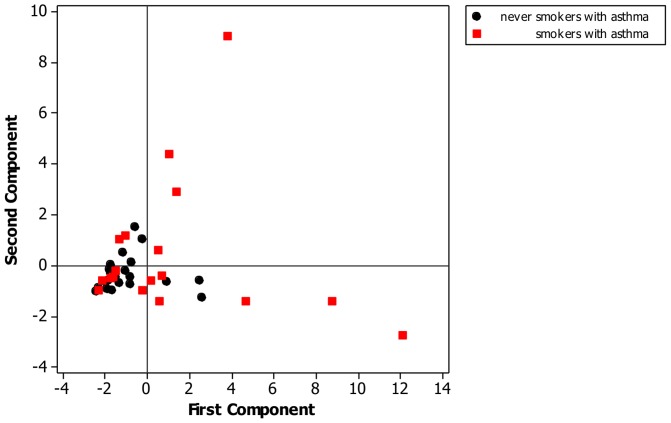
Principal component analysis plot of pre dexamethasone sputum supernatant cytokines (IL1β, 1RA, 2R, 6, 7, 13, 17, IFNα, GM-CSF, CCL2, 3, 4 & 5, CXCL8, 9 & 10). Principal component 1 represents 52% of variance in data, Principal component 2 21%. Based on examination of the component loadings (Table 3) we interpret principal component 1 as being principally driven by the sputum supernatant cytokines IL1β, 2R, 7, 12, 13, 17 & GM-CSF principal component 2 to be driven by sputum supernatant IL-6 and the chemokines CXCL9 & 10 & CCL 2.

Principal component analysis was performed using the correlation matrix from the pre dexamethasone sputum dataset. The decision on number of principal components present was guided by inspection of the scree plot requirement for eigenvalues of greater than 1. Principal component loadings were employed to derive a qualitative description of the sputum cytokine profiles with reference to patient group (figures 1 and S1). Interpretation of principal components was guided by inspection of the derived loadings leading to a description based on the dominant contributing variables. Analysis was performed on SAS v 9.1 (TS1M3) for Windows (SAS Institute Inc., NC, USA) and MINITAB 15 (Minitab Inc. State College, PA, USA).

## Results

Fifty three patients with asthma (22 current smokers, 21 never smokers and 10 ex-smokers) agreed to participate in the study and twenty smokers, nine ex-smokers and seventeen never smokers with asthma were compliant with the oral corticosteroid trial (confirmed by serum cortisol). Of these, eighteen current smokers, nine ex-smokers and sixteen never smokers with asthma provided a satisfactory sputum sample at both visits.

### Baseline Demographics

The groups were generally well balanced (Table 1). However the smoker and ex-smoker groups were prescribed higher daily inhaled corticosteroid doses and had higher ACQ scores. The bronchodilator response of the smoker group was lower compared to never-smokers with asthma. Smokers with asthma had reduced exhaled nitric oxide levels compared to both ex-smokers and never smokers with asthma. Sputum cellular profiles were equivalent across the three groups with no neutrophilia or eosinophilia evident.

**Table 1:** Baseline demographics of participants (n = 53).

	Smokers with asthma (n = 22)	Ex-Smokers with asthma (n = 10)	Never-Smokers with asthma (n = 21)
**Age** (years)	46.6 (6.7)	49.8 (9.0)	42.5 (10.0)
**Gender** (F:M)	12∶10	5∶5	11∶10
**BMI** (kg/m^2^)	26.6 (6.0)	31.2 (5.3)	28.9 (5.1)
**Asthma Duration** (years)	22.1 (15.9)	24.6 (15.9)	28.6 (15.0)
**Atopy** (yes/no/unknown)	10/6/6	4/4/2	15/3/3
**Pack years smoked** Median (IQR)	26.5 (15, 35)	24 (20, 30)	
**Current cigarettes/day** Median (IQR)	17.5 (15, 20)		
**Duration of smoking cessation** (years)		7.7 (4.5)	
**Inhaled steroid** (mcg/day; beclometasone equivalent)Median (IQR)	**800** * (800, 1600)	1000 (800, 2000)	800 (750, 1000)
**ACQ Score** (0 to 6)	**2.2** * (0.9)	2.3 (0.7)	1.5 (0.8)
**Pre BD FEV_1_** (% predicted)	73.6 (18.5)	79.7 (24.1)	73.3 (15.3)
**Pre BD PEF** (% predicted)	81.7 (20.8)	85.4 (24.7)	85.8 (19.1)
**FEV_1_ BD response** median (IQR)	16.4 (13.8, 23.3)	20.0 (13.0, 35.9)	28.5 (14.0, 37.5)
**Pre dexamethasone Fe_NO50_** (ppb) median (IQR)	**11.1** * (3.6, 13.5)	19.8 (15.8, 43.5)	32.8 (17.7, 73.2)
**Total cell count** (10^6^) median (IQR)	7.4 (3.4, 12.3)	10.3 (5.2, 15.3)	5.3 (3.8, 7.8)
**Sputum eosinophils** (% of total) median (IQR)	0.4 (0, 1)	1.0 (0.1, 5)	0.3 (0, 2)
**Sputum neutrophils** (% of total) median (IQR)	34 (24, 56)	37 (22, 63)	24 (10.5, 41)

Data presented as means with standard deviation (SD) except where indicated.

* p≤0.05 relative to never smokers with asthma.

Abbreviations: ACQ; asthma control questionnaire, BD; bronchodilator, BMI; body mass index, mcg;

microgramme, FEV_1_; forced expiratory volume in 1 sec, IQR; interquartile range, PEF; peak expiratory flow.

### Lung Function Response to Dexamethasone

The never-smokers with asthma group made a significant within-group improvement in lung function in response to oral dexamethasone (FEV_1_ change; 173 ml (95% CI: 10, 336), p = 0.039), in contrast to current smokers with asthma (smokers; 32 ml (−115, 178), p = 0.657). The response of ex-smokers with asthma was variable and overlapped that observed in the never-smokers with asthma (ex-smokers; 257 ml (−154, 667), p = 0.188). No statistically significant relationship was evident between lung function response to oral dexamethasone and either time since cessation of smoking or total pack years in ex-smokers with asthma (data not shown).

### Sputum Cell Change in Response to Dexamethasone

The proportion of eosinophils in samples from smokers with asthma changed in response to oral dexamethasone and was equivalent to that observed in non-smokers with asthma (smokers change −0.4% (95% CI −0.8, 0.0), non-smokers −0.2% (−2.0, 0.0), p = 0.430). Smokers with asthma also demonstrated a trend to a reduction in sputum neutrophil percentage (change −12.0% (−25.01, 1.99), p = 0.081). However no difference was evident when compared to the neutrophil change observed in non-smokers (Table 2).

**Table 2:** Sputum profiles changes in response to dexamethasone.

	Smokers with asthma	Ex-Smokers with asthma	Never-Smokers with asthma
	(n = 18)	(n = 9)	(n = 16)
Δ total viable cell count 10*6 cells	0.08 (0.36)	0.23 (0.77)	−0.11 (0.54)
**Δ eosinophil %**	−0.69 (2.87)	−2.82 (3.63)	−1.26* (2.48)
**Δ neutrophil %**	−7.2 (21.3)	4.1 (28.1)	0.6 (32.7)
**Δ macrophages %**	−1.3 (23.4)	−5.7 (18.9)	−7.4 (25.0)
**Δ lymphocytes %**	−0.02 (0.67)	0.00 (0.05)	−0.26 (0.64)
**Δ bronchial epithelial cells %**	9.5* (16.2)	4.3 (19.5)	8.0 (20.5)

Data presented as mean (SD). **Δ**; change. *p<0.05 (within group change).

### Sputum Supernatant Cytokines

Expression levels were below the manufacturers recommended minimum detection level in the majority of subjects for a number of cytokines (IL2, 4, 10, 15, TNFα, CCL11 and IFNγ (see table S1 for detailed description)). Therefore significance tests for these cytokines will not be presented and results for these cytokines were not employed in multivariate analysis. However the remainder of the cytokines and chemokines tested were detectable in the majority of subjects. The summary data for all sputum supernatant cytokines tested (including those with median levels below the manufacturers recommended minimum level) are presented in Table 3.

**Table 3:** Sputum supernatant cytokine concentrations before and after two weeks of oral dexamethasone.

	Smokers with asthma		Ex-smokers with asthma		Never smokers with asthma	
	Pre-steroid (n = 20)	Post-steroid (n = 18)	Pre-steroid (n = 10)	Post-steroid (n = 9)	Pre-steroid (n = 21)	Post-steroid (n = 16)
**IL-1RA**	12480 (3475, 18561)	**16140** 2 (4208, 23359)	5206 (3175, 8231)	12168 (6445, 17519)	3449 (1952, 8641)	4838 (2486, 8711)
**IL-1β**	19.4 (9.0, 34.4)	**28.9** 3 (9.0, 84.0)	13.5 (9.0, 47.4)	62.4 (19.4, 94.8)	9.0 (9.0, 22.0)	**18.1** 4 (9.0, 41.2)
**IL-2**	3.0 (3.0, 6.7)	3.0 (3.0, 11.0)	3.0 (3.0, 3.8)	3.0 (3.0, 14.3)	3.0 (3.0, 3.0)	3.0 (3.0, 3.0)
**IL-2R**	106 (12, 297)	132 (12, 860)	164 (91.3, 577)	113 (26.0, 1036)	40.2 (12.0, 139)	60.3 (12.0, 540)
**IL-4**	2.5 (2.5, 2.5)	4.6 (2.5, 22.2)	2.5 (2.5, 27.7)	2.5 (2.5, 27.7)	2.5 (2.5, 2.5)	2.5 (2.5, 8.6)
**IL-5**	4.7 (1.5, 12.1)	6.5 (1.5, 27.4)	3.5 (2.7, 8.5)	3.8 (3.5, 26.3)	3.1 (1.5, 4.4)	3.8 (1.5, 10.7)
**IL-6**	**34.4** 1 (11.1, 83.5)	**24.2** 2 (15.7, 99.5)	34.9 (13.0, 152)	16.0 (4.3, 63.1)	8.1 (4.0, 11.3)	7.3 (2.2, 22.8)
**IL-7**	**28.5** 1 (13.8, 72.2)	22.6 (7.7, 69.9)	36.2 (12.1, 60.9)	29.3 (5.0, 69.3)	16.3 (5.0, 21.7)	12.6 (3.3, 39.2)
**IL-10**	2.5 (2.5, 9.8)	5.6 (2.5, 12.7)	2.5 (2.5, 6.7)	2.5 (2.5, 13.9)	2.5 (2.5, 2.5)	2.5 (2.5, 7.1)
**IL-12**	**30.6** 1 (12.6, 49.6)	41.5 (19.1, 123)	23.6 (10.9, 59.8)	33.3 (17.4, 124)	15.5 (7.7, 23.1)	18.4 (7.2, 55.6)
**IL-13**	29.4 (20.5, 48.0)	45.5 (20.2, 102)	29.9 (22.5, 59.0)	35.3 (25.3, 115)	24.4 (20.5, 30.0)	25.8 (21.0, 69.0)
**IL-15**	11.0 (5.0, 50.9)	16.5 (5.0, 115)	12.5 (5.0, 81.7)	19.0 (5.0, 157)	5.0 (5.0, 5.0)	5.0 (5.0, 71.1)
**IL-17**	44.9 (8.0, 163)	**116** 3 (8.0, 314)	36.3 (8.0, 171)	86.3 (8.0, 354)	8.0 (8.0, 22.3)	18.2 (8.0, 202)
**GM-CSF**	21.5 (7.5, 76.4)	**56.4** 3 (9.3, 151)	20.1 (5.5, 103)	38.6 (22.4, 209)	17.0 (6.6, 28.9)	31.7 (7.5, 105)
**IFN-α**	24.5 (20.8, 55.5)	**58.5** 3 (20.8, 141)	24.5 (19.8, 87.9)	48.1 (27.9, 143)	20.8 (15.7, 32.6)	31.2 (17.8, 78.9)
**IFN-γ**	2.5 (2.5, 13.7)	6.2 (2.5, 48.2)	2.5 (2.5, 26.4)	2.5 (2.5, 70.4)	2.5 (2.5, 2.5)	2.5 (2.5, 27.2)
**TNF-α**	2.5 (2.5, 8.9)	4.1 (2.5, 11.9)	2.5 (2.5, 7.0)	11.8 (2.5, 15.7)	2.5 (2.5, 2.5)	2.5 (2.5, 7.4)
**CXCL8**	1096 (375, 3357)	1389 (316, 3342)	1715 (460, 6500)	681 (340, 3095)	650 (322, 1226)	400 (192, 1416)
**CXCL9**	128 (44.3, 242)	73.6 (12.0, 237)	191 (89, 268)	157 (43, 248)	91.7 (42.7, 154)	60.1 (18.4, 176)
**CXCL10**	52.6 (31.6, 105)	15.2 (7.1, 32.0)	193 (51.5, 328)	16.4 (9.5, 32.3)	59.8 (25.7, 95.0)	16.3 (5.9, 64.6)
**CCL2**	298 (159, 413)	345 (180, 502)	317 (188, 589)	269 (148, 393)	193 (128, 229)	184 (111, 349)
**CCL3**	26.9 (16.1, 71.7)	41.9 (16.9, 107)	45.4 (27.3, 92.0)	82.6 (20.7, 163)	20.8 (17.4, 30.6)	26.1 (17.4, 59.4)
**CCL4**	30.1 (19.0, 92.8)	47.1 (19.9, 124)	128 (40.3, 372)	59.1 (30.8, 145)	27.8 (17.1, 40.9)	**36.5** 4 (14.6, 97.0)
**CCL5**	42.8 (32.0, 66.3)	29.3 (19.4, 56.8)	58.4 (35.0, 110)	31.1 (18.5, 66.4)	37.3 (22.4, 45.8)	32.5 (7.5, 48.9)
**CCL11**	2.5 (2.5, 7.8)	2.5 (2.5, 12.0)	3.8 (2.5, 6.0)	2.5 (2.5, 6.2)	2.5 (2.5, 2.5)	2.5 (2.5, 2.5)

Data presented as median (IQR). All results pg/ml.

Results with superscript numerals had significance tests <0.05. Specifically,

1 pre-dexamethasone smokers vs. never-smokers with asthma,

2 post-dexamethasone smokers vs. never smokers with asthma,

3 pre-dexamethasone vs. post-dexamethasone smokers with asthma,

4 pre-dexamethasone vs. post-dexamethasone never smokers with asthma.

Cytokine abbreviations: CXC; CXC chemokine motif ligand, CCL; CC chemokine motif ligand, GM-CSF; granulocyte macrophage colony-stimulating factor, IFN; interferon IL; interleukin, TNF; tumor necrosis factor.

Given the difference in baseline inhaled corticosteroid doses, analysis incorporating adjustment of sputum cytokine results for regular inhaled corticosteroid dose was performed to assess the potential impact. Adjustment for baseline inhaled corticosteroid dosage did not reduce any of the differences evident and strengthened some (e.g. IL12, IL17). Based on this finding we present the unadjusted results.

### Pre Dexamethasone Sputum Supernatant Cytokines

Smokers with asthma demonstrated elevated concentrations of a number of sputum supernatant cytokines compared to never smokers with asthma (Table 3). Specifically, smokers with asthma had raised IL-6 (p<0.001), IL-7 (p = 0.033) and IL-12 (p = 0.042). IL1RA (p = 0.055), IL-17 (p = 0.072) and CCL2 (p = 0.083) demonstrated a tendency to a significantly higher concentration in smokers with asthma. Several sputum cytokines also appeared to be raised in ex-smokers with asthma compared to never smokers with asthma (see Table 3 and table S2).

Principal component analysis employing the majority of the pre dexamethasone sputum supernatant cytokines was performed using the data from smokers and never smokers with asthma alone. This suggested a three component solution explaining more than 88% of the variance in the data (Table 4). The first two components explained 73% of the variance (principal component (PC) 1 52% and PC2 21%). Loadings to PC1 were generally low with none above 0.4 whilst there were several variables loading at greater than 0.4 to PC2. The majority of the variance for PC1 and 2 appeared to be driven by the current smokers with asthma (Figure 1). Analysis including the data for ex-smokers was also performed (see figure S1).

**Table 4:** Principal component analysis of pre dexamethasone sputum supernatant cytokines.

	PC1	PC2	PC3
	(57% total variance)	(21% total variance)	(15% total variance)
**IL-1RA**	0.105	0.169	−0.184
**IL-2R**	0.326	−0.063	0.080
**IL-6**	0.082	0.456	−0.012
**IL-7**	0.322	0.013	0.100
**IL-12**	0.326	−0.104	−0.003
**IL-13**	0.312	−0.164	0.033
**IL-17**	0.316	−0.154	0.015
**GM-CSF**	0.310	−0.178	0.054
**IFN-α**	0.321	−0.130	0.054
**CXCL8**	0.010	0.151	−0.588
**CXCL9**	0.155	0.408	0.199
**CXCL10**	0.074	0.436	0.228
**CCL2**	0.131	0.394	−0.046
**CCL3**	0.246	0.033	−0.372
**CCL4**	0.106	0.137	−0.549
**CCL5**	0.224	0.278	0.241

### Sputum Supernatant Cytokines-dexamethasone Response

Few statistically significant within-group changes were evident in response to oral dexamethasone. Smokers with asthma demonstrated an increase in sputum IFNα of 53.5 pg/ml (95% CI 12.1, 94.8), p = 0.014, and in IL-17 of 235.3 pg/ml (83.4, 387.2), p = 0.012. A similar but non-significant trend was also evident for these cytokines in never smokers with asthma (IFNα; 24.7 pg/ml (−4.0, 53.5), p = 0.087, IL-17; 66.4 pg/ml (−8.7, 141.4), p = 0.079). An increase in CCL4 was evident in the never smokers with asthma (31.6 pg/ml (4.6, 58.5), p = 0.03). Some sputum cytokines also appeared to change in ex-smokers with asthma response to dexamethasone (see text S1).

In an attempt to gain insights into the relationship between corticosteroid resistance in airway responses and airway cytokines we examined correlations between CCL4, IFNα and IL-17 and change in FEV_1_% predicted and ACQ score. However no significant correlations were evident (data not shown).

### Plasma Cytokines

Several differences were evident when both groups were compared (Table 5 (and Table S3 for description of samples below manufacturers recommended minimum level). In general, the current smoker group had lower plasma cytokines, with significantly reduced median plasma concentrations of IL1RA (p = 0.019), IL-12 (p = 0.046), IL-13 (p = 0.003) and GM-CSF (p = 0.021).

**Table 5:** Plasma cytokine concentrations before and after two weeks of oral dexamethasone.

	Smokers with asthma		Never smokers with asthma	
	Pre-steroid	Post-steroid	Pre-steroid	Post-steroid
	(n = 19)	(n = 18)	(n = 20)	(n = 17)
**IL-1RA**	**209** 1 (160, 252)	**142** 2 (114, 185)	247 (220, 284)	**179** 4 (160, 215)
**IL-1β**	9.0 (9.0, 20.1)	9.0 (9.0, 11.4)	9.0 (9.0, 17.3)	9.0 (9.0, 19.7)
**IL-2**	3.0 (3.0, 3.0)	3.0 (3.0, 3.0)	3.0 (3.0, 6.6)	3.0 (3.0, 8.0)
**IL-2R**	294 (165, 355)	**177** 3 (128, 223)	251 (211, 306)	**195** 4 (161, 233)
**IL-4**	9.7 (6.3, 11.3)	**6.3** 3 (2.5, 9.3)	10.5 (8.7, 12.7)	**7.3** 4 (5.3, 11.7)
**IL-5**	1.5 (1.5, 1.5)	1.5 (1.5, 1.5)	1.5 (1.5, 1.5)	1.5 (1.5, 1.5)
**IL-6**	1.5 (1.5, 1.5)	1.5 (1.5, 1.5)	1.5 (1.5, 3.3)	1.5 (1.5, 5.4)
**IL-7**	16.3 (5.0, 18.0)	**5.0** 2,3 (5.0, 14.7)	19.7 (14.7, 25.2)	15.4 (12.6, 20.5)
**IL-10**	2.5 (2.5, 2.5)	2.5 (2.5, 2.5)	2.5 (2.5, 2.5)	2.5 (2.5, 2.5)
**IL-12**	**69.1** 1 (46.0, 76.1)	**58.1** 3 (39.1, 65.8)	70.9 (63.6, 86.1)	**53.2** 4 (47.8, 59.9)
**IL-13**	**19.5** 1 (17.4, 21.5)	**19.5** 2 (17.2, 21.5)	21.5 (19.7, 23.4)	21.5 (19.5, 26.3)
**IL-15**	5.0 (5.0, 5.0)	5.0 (5.0, 5.0)	5.0 (5.0, 5.0)	5.0 (5.0, 11.9)
**IL-17**	22.6 (8.0, 47.7)	8.0 (8.0, 40.5)	33.4 (16.3, 56.2)	28.3 (8.0, 43.1)
**GM-CSF**	**6.6** 1 (5.1, 13.0)	10.3 (4.5, 12.1)	10.3 (10.3, 26.1)	10.3 (6.6, 25.2)
**IFN-α**	31.2 (24.5, 34.3)	**29.5** 2 (24.5, 34.3)	32.7 (28.3, 39.4)	34.3 (31.2, 40.2)
**IFN-γ**	2.5 (2.5, 2.5)	2.5 (2.5, 2.5)	2.5 (2.5, 2.5)	2.5 (2.5, 2.5)
**TNF-α**	2.5 (2.5, 2.5)	2.5 (2.5, 2.5)	2.5 (2.5, 2.5)	2.5 (2.5, 2.5)
**CXCL8**	5.1 (3.6, 6.8)	**4.3** 3 (2.6, 5.0)	6.1 (2.4, 8.0)	**4.0** 4 (1.5, 6.2)
**CXCL9**	12.0 (12.0, 12.0)	12.0 (12.0, 12.0)	12.0 (12.0, 14.6)	12.0 (12.0, 12.0)
**CXCL10**	10.0 (8.1, 17.0)	**2.5** 2, 3 (2.5, 5.4)	14.5 (10.6, 18.5)	**6.4** 4 (2.5, 10.7)
**CCL2**	175 (116, 259)	**117** 3 (86, 184)	165 (147, 238)	122 (98.5, 208)
**CCL3**	24.1 (19.1, 25.7)	**20.8** 2 (19.5, 22.4)	24.1 (24.1, 27.3)	24.1 (22.4, 27.3)
**CCL4**	27.8 (24.4, 31.3)	**24.4** 2,3 (21.4, 30.1)	31.3 (27.0, 35.6)	30.1 (27.2, 32.7)
**CCL5**	4639 (3653, 6269)	**3273** 3 (2852, 4230)	6387 (4245, 9545)	**4773** 4 (3383, 5537)
**CCL11**	88.8 (50.6, 130)	**149** 3 (91.0, 210)	75.4 (42.3, 86.7)	**101** 4 (81.4, 126)

Data presented as median (IQR). All results pg/ml.

Results with superscript numerals had significance tests <0.05. Specifically,

1 pre-dexamethasone smokers vs. never smokers with asthma,

2 post-dexamethasone smokers vs. never smokers with asthma,

3 pre- vs. post-dexamethasone smokers with asthma,

4 pre- vs. post-dexamethasone never smokers with asthma.

Following the oral dexamethasone trial a number of plasma cytokines were found to be significantly lower in smokers with asthma (compared to never smokers with asthma) (Table 5). These were IL1RA (p = 0.028), IL-7 (p = 0.005), IL-13 (p = 0.012), IFNα (p = 0.028), CXCL10 (p = 0.007), CCL3 (p = 0.011) and CCL4 (p = 0.007).

## Discussion

Our findings demonstrate a clear impact of current smoking on sputum cytokine profiles in patients with stable chronic asthma. An additional important finding is that the cytokine profiles in both current and former smokers with asthma appear to be insensitive to high dose oral corticosteroids. We believe the presented results are of importance for several reasons. Cigarette smoke is known to be a potent immunomodulator [25] and there is ample evidence for an adverse impact of smoking on asthma. For example, airway epithelial appearances in asthma are known to be altered by current smoking [18], [26] and current smokers with asthma show attenuated responses to both inhaled and oral corticosteroids [1], [2], [3], [4], [5], [6], [7], [8], [9]. However little is known about the mechanisms by which current cigarette smoking drives this altered phenotype. Whilst asthma is typically described as a prototypical Th2 disease, it has more recently been suggested to be more accurate to view asthma as a heterogeneous condition given the disappointing responses observed to immuno-therapies targeted against key Th2 cytokines [27], [28]. Current smokers with asthma are common in clinical practice and can be argued to represent a further ‘subtype’ given their clinical behavior and altered airway cytokine profiles compared to never smokers with asthma.

We present evidence that current smokers with asthma have increased sputum supernatant concentrations of IL6, 7 and 12 and an absence of sputum neutrophilia. The lack of improvement in lung function to oral dexamethasone in this cohort also suggests the presence of corticosteroid resistant airways disease. We used oral dexamethasone as it allowed detection of suppression of serum cortisol as an indicator of compliance with the corticosteroid trial. Therefore we are confident that we are not observing a reduced lung function response due to poor adherence.

Our results demonstrate that IL6 is elevated in both current and ex-smokers with asthma and this cytokine also failed to respond to oral dexamethasone. Of interest is our previous observation that treatment with low dose theophylline is associated with a fall in sputum supernatant IL-6 concentration in current smokers with asthma (unpublished observations and [24]). IL-6 is a pleiotropic cytokine which has an important role in Th-17 cell development and is elevated in viral exacerbations of airways disease; a state recognized to be relatively corticosteroid resistant [29], [30]. Bronchoalveolar lavage samples from corticosteroid resistant non-smokers with asthma have also demonstrated increased concentrations of IL-6 [31] and therefore IL-6 may be promoting corticosteroid resistant inflammation via promotion of Th17 development and activation. A second alternative explanation is that our results reflect unchallenged promotion of IL-6 production via β2 agonists due to a loss of corticosteroid sensitivity in the IL-6 gene [30]. A third potential explanation is the observed increased concentration of sputum supernatant IL-6 reflects increased local production in an attempt to suppress inflammation [32], [33].

Whilst our study only includes a small number of ex-smokers with asthma, we provide some evidence that ex-smokers with asthma have ongoing inflammatory signaling despite smoking cessation and perhaps a differing sputum cytokine profile from current smokers with asthma. The reasons why this pattern exists in ex-smokers with asthma is not entirely clear but may reflect a persistent permissive effect of smoking on cytokine expression at a transcriptional level.

Few sputum supernatant cytokine concentrations were observed to change significantly in response to oral dexamethasone. Interestingly, IFNα and IL-17 increased after oral dexamethasone in current smokers with asthma. Impaired IFNα production has been linked to the development of viral-induced exacerbations of asthma [34]. Little is known about the response of current smokers with asthma to viral infections and their subsequent response to corticosteroids. However our results suggest that a corticosteroid sensitive and potentially appropriate response is present in this group. In contrast the observed increase in IL-17 suggests a potential mechanism for the poor lung function response to corticosteroids of smokers with asthma. The observed dexamethasone related increase in sputum supernatant IL-17 is of interest given the known links between IL-17 and corticosteroid insensitive inflammation and suggests a potential role for IL-17 and perhaps Th17 cells in the altered corticosteroid responses of smokers with asthma [35].

Current smokers with asthma had significantly lower concentrations of plasma IL-1RA, 12, 13 and GM-CSF compared to non smokers with asthma. This reduction in plasma cytokines in contrast to elevation of a number of airway cytokines suggests that current smokers with asthma, in contrast to COPD, do not suffer from a generalized systemic inflammation due to an ‘overspill’ of pulmonary inflammation. The disparity between the sputum and systemic cytokine concentrations also lead us to suggest that future studies should aim to measure cytokines from airway samples in addition to peripheral blood in asthma as valuable insights may otherwise be missed.

Whilst this is the first study providing an assessment of a wide range of airway cytokines in smokers and ex-smokers with asthma, we recruited a relatively small number of participants, particularly ex-smokers with asthma, and our results require corroboration in further studies. Finally we did not examine the cellular source of the cytokines measured in this study therefore our conclusions require validation using airway cell preparations isolated from BAL or sputum and bronchial brushings.

### Conclusions

Current smokers with asthma display alterations of both sputum supernatant and peripheral cytokine profiles when compared to never smokers with asthma. Elevated sputum cytokines in current smokers with asthma are unresponsive to oral corticosteroids and are associated with a poor lung function response. Ex-smokers with asthma may display a differing airway cytokine profile to current smokers with asthma but further work is required to examine this possibility. Given our findings we suggest that ex-smokers with asthma could represent a distinct group and caution against viewing ex-smokers with asthma as automatically equivalent to current smokers with asthma. Finally future studies should endeavor to determine the airway cellular sources of the elevated cytokine concentrations in current and ex-smokers with asthma and to evaluate how cigarette smoking induced cytokine changes could be driving relatively corticosteroid resistant airway inflammation as such efforts will support development of novel targeted therapies for asthma.

## Supporting Information

Figure S1 **Principal component analysis plot of pre dexamethasone sputum supernatant cytokines (IL1β, 1RA, 2R, 6, 7, 13, 17, IFNα, GMCSF, CCL2, 3, 4 & 5, CXCL8, 9 & 10) including data from ex-smokers with asthma.** Principal component 1 represents 57% of variance in data, Principal component 2 16%. Based on examination of the component loadings we interpret principal component 1 as being principally driven by the sputum supernatant cytokines IFNα, IL1β, 2R, 7, 12, 13, 17 & GMCSF and principal component 2 IL6, 8, CCL2, 4 and CXCL10.
(TIF)

Table S1 **Number and percentage of sputum supernatant samples per cytokine (grouped according to smoking history) below the manufacturer’s recommended lower limit of detection.**
(DOCX)

Table S2 **Comparison of smokers with never smokers with asthma pre and post dexamethasone.**
(DOCX)

Table S3 **Number and percentage of serum samples per cytokine (grouped according to smoking history) below the manufacturer’s recommended lower limit of detection.**
(DOCX)

Text S1 **Few statistically significant within-group changes were evident in the ex-smokers with asthma group in response to oral dexamethasone.** However ex-smokers with asthma did demonstrated a reduction in CCL5 (−21.7 pg/ml (−41, −3), p = 0.03) and CXCL10 (−147.7 pg/ml (−251, −45), p = 0.01).
(DOCX)
