# Supplementary figures and images for: Smoking in Asthma Is Associated with Elevated Levels of Corticosteroid Resistant Sputum Cytokines—An Exploratory Study
Source: PLoS One. 2013 Aug 9;8(8):e71460. doi: 10.1371/journal.pone.0071460 (PMC3739804; doi:10.1371/journal.pone.0071460)

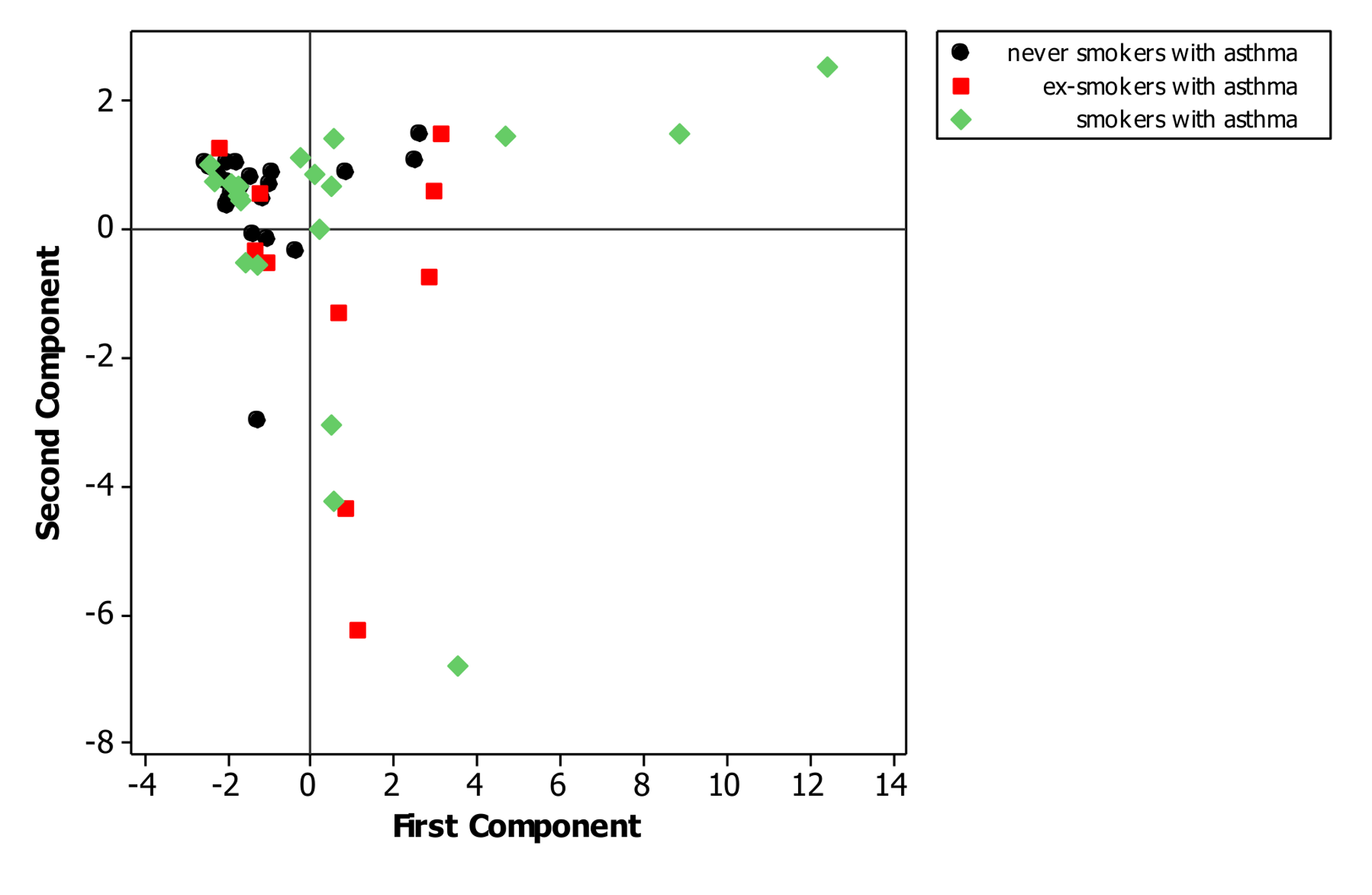

Supplement: Figure S1 — Principal component analysis plot of pre dexamethasone sputum supernatant cytokines (IL1β, 1RA, 2R, 6, 7, 13, 17, IFNα, GMCSF, CCL2, 3, 4 & 5, CXCL8, 9 & 10) including data from ex-smokers with asthma. Principal component 1 represents 57% of variance in data, Principal component 2 16%. Based on examination of the component loadings we interpret principal component 1 as being principally driven by the sputum supernatant cytokines IFNα, IL1β, 2R, 7, 12, 13, 17 & GMCSF and principal component 2 IL6, 8, CCL2, 4 and CXCL10. (TIF) [file pone.0071460.s001.tif]
