# Supplementary material for: Smoking in Asthma Is Associated with Elevated Levels of Corticosteroid Resistant Sputum Cytokines—An Exploratory Study
Source: PLoS One. 2013 Aug 9;8(8):e71460. doi: 10.1371/journal.pone.0071460 (PMC3739804; doi:10.1371/journal.pone.0071460)
Supplement: Table S1 — Number and percentage of sputum supernatant samples per cytokine (grouped according to smoking history) below the manufacturer’s recommended lower limit of detection. (DOCX) [file pone.0071460.s002.docx]

*Table S1.* Number and percentage of sputum supernatant samples per cytokine (grouped according to smoking history) below the manufacturer’s recommended lower limit of detection

|  | Smokers with asthma | | Ex-smokers with asthma | | Never smokers with asthma | |
| --- | --- | --- | --- | --- | --- | --- |
|  | (n) | % | (n) | % | (n) | % |
| **IL-1RA** | 0 | 0.0 | 0 | 0.0 | 0 | 0.0 |
| **IL-1β** | 9 | 40.9 | 5 | 50.0 | 9 | 42.9 |
| **IL-2** | 14 | 63.6 | 8 | 80.0 | 20 | 95.2 |
| **IL-2R** | 6 | 27.3 | 1 | 10.0 | 7 | 33.3 |
| **IL-4** | 18 | 81.8 | 8 | 80.0 | 19 | 90.5 |
| **IL-5** | 8 | 36.4 | 2 | 20.0 | 8 | 38.1 |
| **IL-6** | 0 | 0.0 | 1 | 10.0 | 3 | 14.3 |
| **IL-7** | 3 | 13.6 | 2 | 20.0 | 7 | 33.3 |
| **IL-10** | 14 | 63.6 | 6 | 60.0 | 18 | 85.7 |
| **IL-12** | 2 | 9.1 | 0 | 0.0 | 2 | 9.5 |
| **IL-13** | 0 | 0.0 | 0 | 0.0 | 0 | 0.0 |
| **IL-15** | 10 | 45.5 | 5 | 50.0 | 17 | 81.0 |
| **IL-17** | 9 | 40.9 | 4 | 40.0 | 14 | 66.7 |
| **GM-CSF** | 3 | 13.6 | 2 | 20.0 | 4 | 19.0 |
| **IFN-α** | 1 | 4.5 | 0 | 0.0 | 3 | 14.3 |
| **IFN-γ** | 11 | 50.0 | 7 | 70.0 | 18 | 85.7 |
| **TNF-α** | 16 | 72.7 | 6 | 60.0 | 17 | 81.0 |
| **CXCL8** | 0 | 0.0 | 0 | 0.0 | 0 | 0.0 |
| **CXCL9** | 0 | 0.0 | 0 | 0.0 | 0 | 0.0 |
| **CXCL10** | 0 | 0.0 | 1 | 10.0 | 0 | 0.0 |
| **CCL2** | 0 | 0.0 | 0 | 0.0 | 0 | 0.0 |
| **CCL3** | 0 | 0.0 | 0 | 0.0 | 0 | 0.0 |
| **CCL4** | 0 | 0.0 | 0 | 0.0 | 0 | 0.0 |
| **CCL5** | 0 | 0.0 | 1 | 10.0 | 3 | 14.3 |
| **CCL11** | 12 | 54.5 | 5 | 50.0 | 20 | 95.2 |
