# Supplementary material for: Smoking in Asthma Is Associated with Elevated Levels of Corticosteroid Resistant Sputum Cytokines—An Exploratory Study
Source: PLoS One. 2013 Aug 9;8(8):e71460. doi: 10.1371/journal.pone.0071460 (PMC3739804; doi:10.1371/journal.pone.0071460)
Supplement: Table S2 — Comparison of smokers with never smokers with asthma pre and post dexamethasone. (DOCX) [file pone.0071460.s003.docx]

Table S2*.* Comparison of smokers with never smokers with asthma pre and post dexamethasone

|  | **Ex-smokers with asthma** | | **Never smokers with asthma** | |
| --- | --- | --- | --- | --- |
|  | Pre-steroid | Post-steroid | Pre-steroid | Post-steroid |
|  | (n=10) | (n=9) | (n=21) | (n=16) |
| **IL-1RA** | 5206 (3175, 8231) | 12168 (6445, 17519) | 3449 (1952, 8641) | 4838 (2486, 8711) |
| **IL-1β** | 13.5 (9.0, 47.4) | 62.4 (19.4, 94.8) | 9.0 (9.0, 22.0) | **18.1 ^4^** (9.0, 41.2) |
| **IL-2** | 3.0 (3.0, 3.8) | 3.0 (3.0, 14.3) | 3.0 (3.0, 3.0) | 3.0 (3.0, 3.0) |
| **IL-2R** | **164** **^1^** (91.3, 577) | 113 (26.0, 1036) | 40.2 (12.0, 139) | 60.3 (12.0, 540) |
| **IL-4** | 2.5 (2.5, 27.7) | 2.5 (2.5, 27.7) | 2.5 (2.5, 2.5) | 2.5 (2.5, 8.6) |
| **IL-5** | 3.5 (2.7, 8.5) | 3.8 (3.5, 26.3) | 3.1 (1.5, 4.4) | 3.8 (1.5, 10.7) |
| **IL-6** | **34.9** **^1^** (13.0, 152) | 16.0 (4.3, 63.1) | 8.1 (4.0, 11.3) | 7.3 (2.2, 22.8) |
| **IL-7** | 36.2 (12.1, 60.9) | 29.3 (5.0, 69.3) | 16.3 (5.0, 21.7) | 12.6 (3.3, 39.2) |
| **IL-10** | 2.5 (2.5, 6.7) | 2.5 (2.5, 13.9) | 2.5 (2.5, 2.5) | 2.5 (2.5, 7.1) |
| **IL-12** | 23.6 (10.9, 59.8) | 33.3 (17.4, 124) | 15.5 (7.7, 23.1) | 18.4 (7.2, 55.6) |
| **IL-13** | 29.9 (22.5, 59.0) | 35.3 (25.3, 115) | 24.4 (20.5, 30.0) | 25.8 (21.0, 69.0) |
| **IL-15** | 12.5 (5.0, 81.7) | 19.0 (5.0, 157) | 5.0 (5.0, 5.0) | 5.0 (5.0, 71.1) |
| **IL-17** | 36.3 (8.0, 171) | 86.3 (8.0, 354) | 8.0 (8.0, 22.3) | 18.2 (8.0, 202) |
| **GM-CSF** | 20.1 (5.5, 103) | 38.6 (22.4, 209) | 17.0 (6.6, 28.9) | 31.7 (7.5, 105) |
| **IFN-α** | 24.5 (19.8, 87.9) | 48.1 (27.9, 143) | 20.8 (15.7, 32.6) | 31.2 (17.8, 78.9) |
| **IFN-γ** | 2.5 (2.5, 26.4) | 2.5 (2.5, 70.4) | 2.5 (2.5, 2.5) | 2.5 (2.5, 27.2) |
| **TNF-α** | 2.5 (2.5, 7.0) | 11.8 (2.5, 15.7) | 2.5 (2.5, 2.5) | 2.5 (2.5, 7.4) |
| **CXCL8** | 1715 (460, 6500) | 681 (340, 3095) | 650 (322, 1226) | 400 (192, 1416) |
| **CXCL9** | **191** **^1^** (89, 268) | 157 (43, 248) | 91.7 (42.7, 154) | 60.1 (18.4, 176) |
| **CXCL10** | 193 (51.5, 328) | **16.4** **^3^** (9.5, 32.3) | 59.8 (25.7, 95.0) | 16.3 (5.9, 64.6) |
| **CCL2** | **317** **^1^** (188, 589) | 269 (148, 393) | 193 (128, 229) | 184 (111, 349) |
| **CCL3** | **45.4** **^1^** (27.3, 92.0) | 82.6 (20.7, 163) | 20.8 (17.4, 30.6) | 26.1 (17.4, 59.4) |
| **CCL4** | **128** **^1^** (40.3, 372) | 59.1 (30.8, 145) | 27.8 (17.1, 40.9) | **36.5 ^4^** (14.6, 97.0) |
| **CCL5** | 58.4 (35.0, 110) | **31.1** **^3^** (18.5, 66.4) | 37.3 (22.4, 45.8) | 32.5 (7.5, 48.9) |
| **CCL11** | 3.8 (2.5, 6.0) | 2.5 (2.5, 6.2) | 2.5 (2.5, 2.5) | 2.5 (2.5, 2.5) |

Data presented as median (IQR). All results pg/ml.

Results with superscript numerals had significance tests <0.05. Specifically, ^1^ pre-dexamethasone ex-smokers vs. never-smokers with asthma, ^2^post-dexamethasone ex vs. never smokers with asthma, ^3^pre-dexamethasone vs. post-dexamethasone ex-smokers with asthma, ^4^pre-dexamethasone vs. post-dexamethasone never smokers with asthma.
