# Supplementary material for: Smoking in Asthma Is Associated with Elevated Levels of Corticosteroid Resistant Sputum Cytokines—An Exploratory Study
Source: PLoS One. 2013 Aug 9;8(8):e71460. doi: 10.1371/journal.pone.0071460 (PMC3739804; doi:10.1371/journal.pone.0071460)
Supplement: Table S3 — Number and percentage of serum samples per cytokine (grouped according to smoking history) below the manufacturer’s recommended lower limit of detection. (DOCX) [file pone.0071460.s004.docx]

*Table S3.* Number and percentage of serum samples per cytokine (grouped according to smoking history) below the manufacturer’s recommended lower limit of detection

|  | Never smokers with asthma | | Smokers with asthma | |
| --- | --- | --- | --- | --- |
|  | (n) | % | (n) | % |
| **IL-1RA** | 0 | 0.0 | 0 | 0.0 |
| **IL-1β** | 16 | 76.2 | 17 | 77.3 |
| **IL-2** | 15 | 71.4 | 19 | 86.4 |
| **IL-2R** | 0 | 0.0 | 0 | 0.0 |
| **IL-4** | 1 | 4.8 | 2 | 9.1 |
| **IL-5** | 19 | 90.5 | 21 | 95.5 |
| **IL-6** | 14 | 66.7 | 20 | 90.9 |
| **IL-7** | 3 | 14.3 | 7 | 31.8 |
| **IL-10** | 18 | 85.7 | 21 | 95.5 |
| **IL-12** | 0 | 0.0 | 0 | 0.0 |
| **IL-13** | 0 | 0.0 | 2 | 9.1 |
| **IL-15** | 17 | 81.0 | 20 | 90.9 |
| **IL-17** | 3 | 14.3 | 8 | 36.4 |
| **GM-CSF** | 1 | 4.8 | 4 | 18.2 |
| **IFN-α** | 0 | 0.0 | 0 | 0.0 |
| **IFN-γ** | 18 | 85.7 | 19 | 86.4 |
| **TNF-α** | 21 | 100.0 | 22 | 100.0 |
| **CXCL8** | 4 | 19.0 | 3 | 13.6 |
| **CXCL9** | 0 | 0.0 | 0 | 0.0 |
| **CXCL10** | 0 | 0.0 | 0 | 0.0 |
| **CCL2** | 0 | 0.0 | 0 | 0.0 |
| **CCL3** | 0 | 0.0 | 0 | 0.0 |
| **CCL4** | 0 | 0.0 | 0 | 0.0 |
| **CCL5** | 0 | 0.0 | 0 | 0.0 |
| **CCL11** | 0 | 0.0 | 1 | 4.5 |
