# Supplementary material for: Smoking in Asthma Is Associated with Elevated Levels of Corticosteroid Resistant Sputum Cytokines—An Exploratory Study
Source: PLoS One. 2013 Aug 9;8(8):e71460. doi: 10.1371/journal.pone.0071460 (PMC3739804; doi:10.1371/journal.pone.0071460)
Supplement: Text S1 — Few statistically significant within-group changes were evident in the ex-smokers with asthma group in response to oral dexamethasone. However ex-smokers with asthma did demonstrated a reduction in CCL5 (−21.7 pg/ml (−41, −3), p = 0.03) and CXCL10 (−147.7 pg/ml (−251, −45), p = 0.01). (DOCX) [file pone.0071460.s005.docx]

**Sputum supernatant cytokines-dexamethasone response**

Few statistically significant within-group changes were evident in the ex-smokers with asthma group in response to oral dexamethasone. However ex-smokers with asthma did demonstrated a reduction in CCL5 (-21.7 pg/ml (-41, -3), p=0.03) and CXCL10 (-147.7 pg/ml (-251, -45), p=0.01).
